# Noninvasive Evaluation of Trop2 Expression Using ^64^Cu-NOTA-Trodelvy-F(ab’)_2_ in Gastric and Pancreatic Cancer

**DOI:** 10.1021/acs.molpharmaceut.5c01223

**Published:** 2025-11-07

**Authors:** Wenpeng Huang, Fangfang Chao, Ruobing Li, Yihan Yang, Jessica C. Hsu, Molly C. DeLuca, Jonathan W. Engle, Xingmin Han, Lei Kang, Weibo Cai

**Affiliations:** † Department of Nuclear Medicine, 26447 Peking University First Hospital, Beijing 100034, China; ‡ Departments of Radiology and Medical Physics, 5228 University of Wisconsin - Madison, Madison, Wisconsin 53705, United States; § Department of Nuclear Medicine, 191599 The First Affiliated Hospital of Zhengzhou University, Zhengzhou, Henan Province 450052, China; ∥ College of Letters and Science, University of Wisconsin − Madison, Madison, Wisconsin 53705, United States

**Keywords:** Trop2, immunoPET, F(ab’)_2_ fragments, gastric cancer, pancreatic cancer

## Abstract

This study aimed to develop and evaluate a ^64^Cu-labeled NOTA-conjugated F­(ab’)_2_ fragment of Trodelvy for ImmunoPET imaging of Trop2 expression in gastric and pancreatic cancer models. Trodelvy was enzymatically digested using IdeS protease to generate F­(ab’)_2_ fragments, which were subsequently conjugated to a NOTA chelator and radiolabeled with ^64^Cu. *In vitro* binding affinity and cellular uptake were assessed in Trop2-positive and Trop2-negative cancer cell lines using flow cytometry, immunofluorescence, and cell binding assays. *In vivo* ImmunoPET imaging and biodistribution studies were conducted in subcutaneous xenograft models. In NCI-N87 xenografts, ^64^Cu-NOTA-Trodelvy-F­(ab’)_2_ showed peak tumor uptake at 12 h postinjection (10.83 ± 2.76%ID/g), significantly higher than in HGC-27 (3.33 ± 1.12%ID/g, *p* = 0.0110). In BxPC3 xenografts, uptake reached 10.00 ± 0.89%ID/g at 12 h, compared to 3.13 ± 0.96%ID/g in AsPC1 (*p* < 0.0001). Tumor-to-heart ratios were significantly improved in Trop2-positive tumors: NCI-N87 (3.53 ± 0.22 vs 0.83 ± 0.34 for intact Trodelvy, *p* = 0.0003) and BxPC3 (3.77 ± 0.07 vs 0.77 ± 0.19, *p* < 0.0001). *Ex vivo* biodistribution at 48 h confirmed high tumor retention (NCI-N87: 4.14 ± 0.44%ID/g; BxPC3: 4.10 ± 0.31%ID/g) and significantly lower uptake in control and IgG-F­(ab’)_2_ groups (*p* < 0.01). Although renal uptake was elevated due to the clearance of antibody fragments, histological analyses showed no signs of off-target toxicity. The rapid tumor targeting and favorable pharmacokinetics of ^64^Cu-NOTA-Trodelvy-F­(ab’)_2_ support its utility as a same-day ImmunoPET imaging agent. This practical and sensitive platform is highly valuable for patient stratification and therapeutic monitoring in Trop2-targeted cancer treatment.

## Graphical abstract

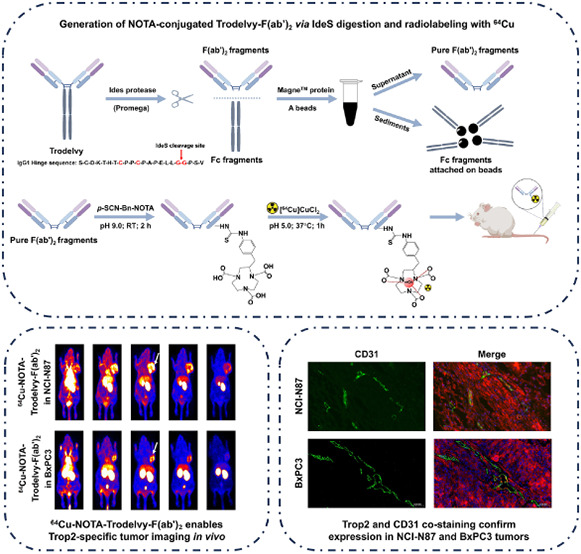

## Introduction

Gastric and pancreatic cancers are among the leading causes of cancer-related mortality worldwide, with current treatment strategies increasingly incorporating immunotherapy and molecularly targeted agents alongside surgical intervention.^1–6^ Improved survival outcomes depend on the development of sensitive tools for early diagnosis and treatment monitoring.^7^ Trop2, a transmembrane glycoprotein involved in tissue development and regeneration, is frequently upregulated in multiple malignancies and correlates with aggressive disease phenotypes and metastatic potential.^8,9^ Trodelvy, an antibody–drug conjugate targeting Trop2, has demonstrated clinical efficacy in various malignancies, including triple-negative breast cancer. However, assessing Trop2 expression and the in vivo biodistribution of such biologics remains challenging due to the lack of noninvasive, quantitative imaging tools to guide patient selection and monitor treatment response.^10–12^

ImmunoPET represents a powerful noninvasive imaging approach to detect Trop2 expression, with strong potential to guide personalized therapy.^13,14^ Conventional imaging agents based on full-length antibodies, however, suffer from limited tissue penetration and prolonged systemic circulation.^15^ In contrast, F­(ab’)_2_ fragments, lacking the Fc regions, exhibit improved pharmacokinetics, including faster blood clearance and reduced immunogenicity, making them well-suited for molecular imaging applications.^16–18^ When radiolabeled with copper-64, which has a half-life compatible with the kinetics of antibody fragments, these probes can enable early, high-contrast imaging.

Trodelvy is currently under active clinical investigation for esophagogastric adenocarcinoma (NCT06123468), while its application in pancreatic cancer remains under preclinical evaluation.^19,20^ This highlights an urgent need for companion diagnostics that can noninvasively assess Trop2 expression and guide therapeutic decisions. Building upon our prior work developing Trop2-targeted imaging agents for gastric, breast, and bladder cancers,^21,22^ we prioritized gastric and pancreatic cancers due to the high prevalence of Trop2 overexpression and its association with adverse prognosis in both diseases, coupled with active clinical development of Trop2-directed therapies, we engineered ^64^Cu-labeled Trodelvy-F­(ab’)_2_ fragments to image Trop2 expression in preclinical gastric and pancreatic cancer models. This approach aims to optimize imaging windows and improve diagnostic accuracy, advancing the translational potential of ^64^Cu-NOTA-Trodelvy-F­(ab’)_2_ for high-mortality cancers with pressing diagnostic gaps and strong clinical relevance for Trop2-targeted therapy.

## Materials and Methods

Comprehensive characterization of ^64^Cu-NOTA-Trodelvy and ^64^Cu-NOTA-Trodelvy-F­(ab’)_2_ was performed using various analytical methods. Trop2 expression in human gastric cancers (NCI-N87, HGC-27) and human pancreatic cancer (BxPC3, AsPC1) cell lines was assessed by flow cytometry and immunofluorescence imaging. Binding affinity and specificity of the radiotracer were evaluated via cell uptake and competitive binding assays.

Athymic Nude-Foxn1nu mice (Envigo) bearing subcutaneous NCI-N87, HGC-27, BxPC3, or AsPC1 tumors were intravenously administered ^64^Cu-NOTA-Trodelvy-F­(ab’)_2_ or ^64^Cu-NOTA-Trodelvy (7.4–11.1 MBq/100 μL). Longitudinal PET imaging was conducted at 1, 4, 12, 24, and 48 h postinjection to assess probe biodistribution and tumor uptake. Detailed experimental protocols are provided in the Supporting Information. All applicable international, national, and/or institutional guidelines for the care and use of animals were followed. The study was approved by the Medical Ethics Committee of the First Affiliated Hospital of Zhengzhou University (2021-KY-1070–002), the Institutional Animal Care and Use Committee at the University of Wisconsin-Madison (#M005630), and the Animal Committee of Peking University First Hospital (No. J2023059).

## Results

### Preparation of ^64^Cu-NOTA-Trodelvy-F­(ab’)_2_

As shown in Figure 1A, the fragmentation of Trodelvy was performed using IdeS protease, which specifically cleaves human antibodies at a single site in the lower hinge region. The Fc fragments were then separated from the F­(ab’)_2_ fragments using Mange Protein A beads and MagneHis Ni particles. Subsequently, the purified F­(ab’)_2_ fragments were conjugated with the NOTA chelator and labeled with copper-64 (Figure S1, Supporting Information). The purified F­(ab’)_2_ fragments have an approximate molecular weight of 100 kDa, while the full-length Trodelvy has a molecular weight of approximately 150–160 kDa, as confirmed by SDS-PAGE analysis (Figure 1B). The presence of a double band under nonreducing conditions likely reflects conformational or glycosylation heterogeneity within the F­(ab’)_2_ domain. HPLC analysis also verified the successful preparation and purification Trodelvy-F­(ab’)_2_ fragments, with the Trodelvy-F­(ab’)_2_ peak eluting later than the full-length Trodelvy antibody peak (Figure 1C).

**Figure 1:**
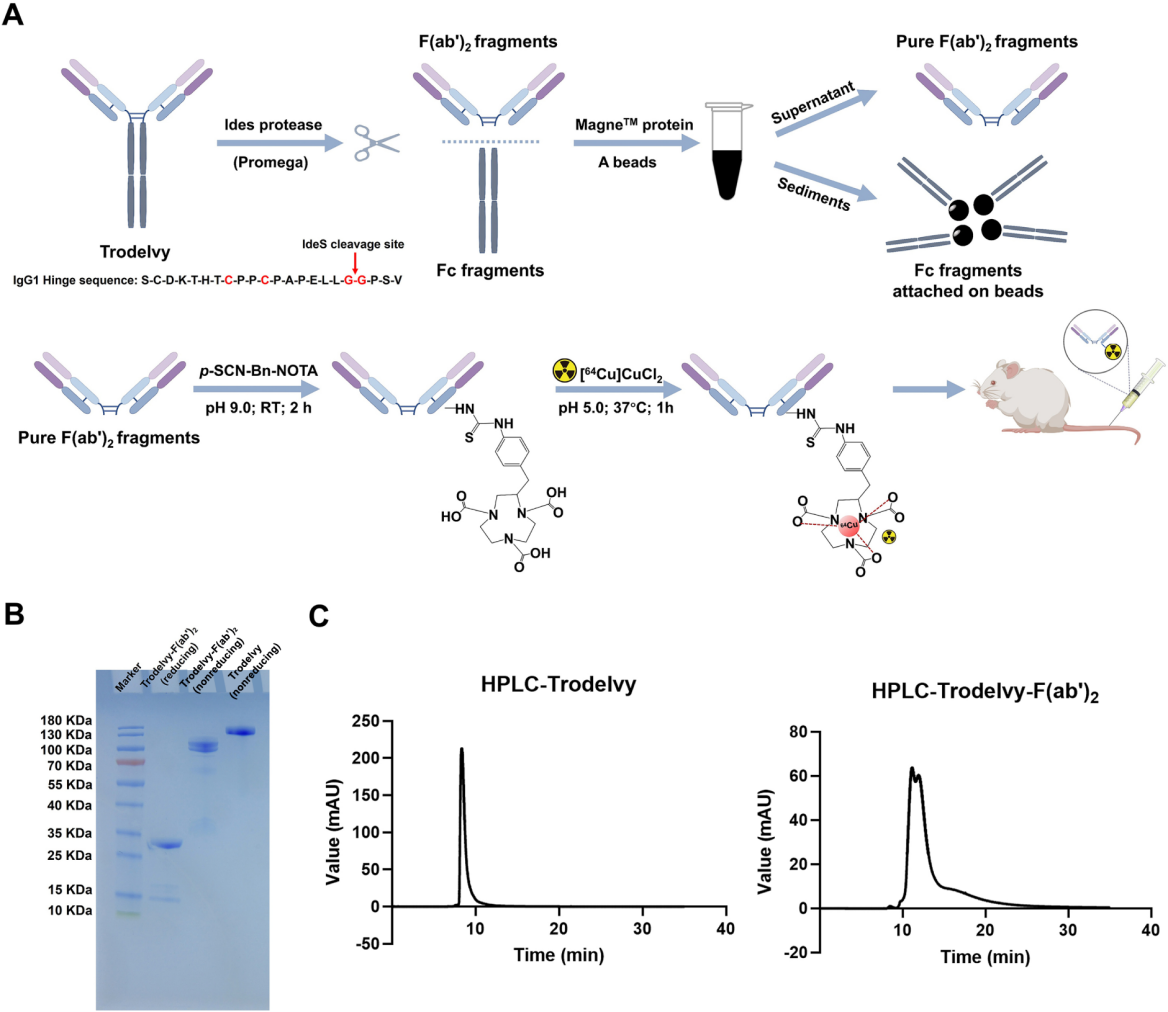
Preparation and characterization of Trodelvy-F­(ab’)_2_ and subsequent conjugation with NOTA for radiolabeling with ^64^Cu. (A) Schematic illustration of the antibody fragmentation and purification workflow, followed by NOTA chelator conjugation and radiolabeling of Trodelvy-F­(ab’)_2_ with the Cu-64 radioisotope. (B) Nonreducing SDS-PAGE and (C) HPLC analyses confirm the successful generation and high purity of Trodelvy fragments.

### Radiosynthesis of ^64^Cu-NOTA-Trodelvy-F­(ab’)_2_

The prepurification radiolabeling efficiency was 82.25 ± 2.41% (Supplementary Figure 1A). The final labeled product, obtained using a PD-10 desalting column, exhibited radiochemical purity above 99% (Supplementary Figure 1B). Stability evaluations revealed that the radiolabeled construct maintained >95% radiochemical integrity after 24 h of incubation in both phosphate-buffered saline and human serum albumin, confirming its robustness for *in vivo* molecular imaging applications (Supplementary Figure 1C and D).

### Cellular Screening and Binding Affinity

Flow cytometry was used to evaluate the relative expression of Trop2 in two human gastric and two pancreatic cancer cell lines using unlabeled Trodelvy, Trodelvy-F­(ab’)_2_, or their NOTA-conjugated counterparts as primary antibodies. Among the gastric cancer cell types, NCI-N87 has high expression of Trop2, while HGC-27 has minimal expression. In terms of pancreatic cancer, BxPC3 has the highest Trop2 expression among the pancreatic cancer lines, with AsPC1 displaying the lowest. The binding affinity of Trodelvy was not affected by the conjugation of the NOTA chelator (Figure 2A).

**Figure 2:**
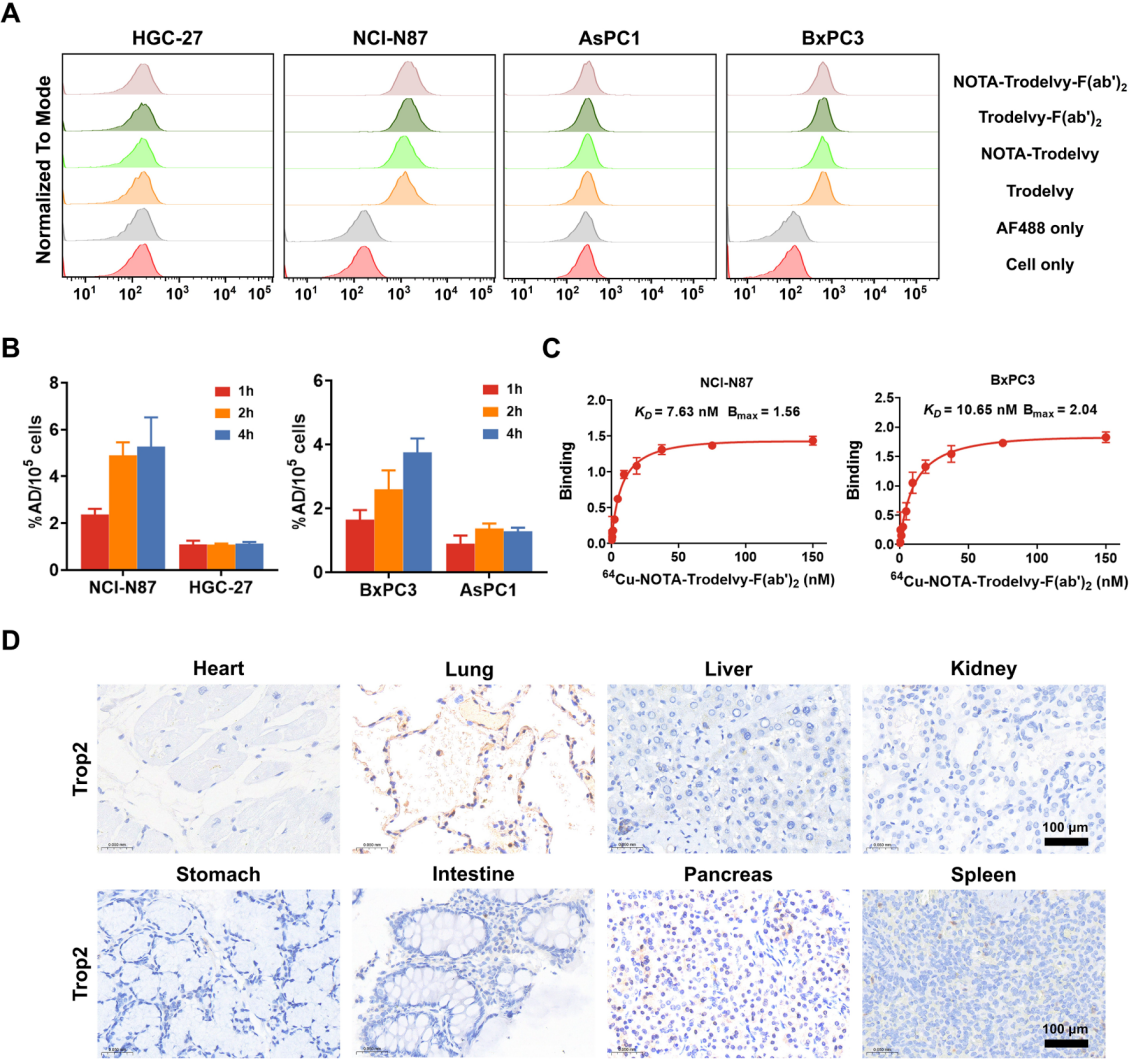
In vitro evaluation of Trop2 expression and binding characteristics of radiolabeled Trodelvy and its fragments. (A) Flow cytometry analysis of Trop2 expression in gastric and pancreatic cancer cell lines using unlabeled Trodelvy, Trodelvy-F­(ab’)_2_, or their NOTA-conjugated counterparts as primary antibodies, followed by detection with Alexa Fluor 488-conjugated rabbit antihuman IgG secondary antibody (Cat# A56021, Thermo Fisher Scientific). (B–C) Binding affinity (*K*_D_) and maximum binding (*B*_max_) of ^64^Cu-NOTA-Trodelvy and ^64^Cu-NOTA-Trodelvy-F­(ab’)_2_ determined by saturation binding assays in NCI-N87 and BxPC3 cells. (D) Immunohistochemical staining of Trop2 expression in selected normal human tissues using antihuman Trop2 antibody (clone EPR20043, Abcam; dilution 1:300).

*In vitro* cellular uptake of ^64^Cu-labeled Trodelvy and Trodelvy-F­(ab’)_2_ was studied in these cancer cell lines and reported as the percentage of the added dose (%AD) per 10^5^ cells. NCI-N87 cells demonstrated a time-dependent increase in ^64^Cu-NOTA-Trodelvy-F­(ab’)_2_ uptake: 2.37 ± 0.20%AD/10^5^ cells at 1 h, 4.90 ± 0.45%AD/10^5^ cells at 2 h, and 5.28 ± 1.02%AD/10^5^ cells at 4 h. Similarly, BxPC3 cells exhibited uptake values of 1.65 ± 0.24%AD/10^5^ cells at 1 h, 2.60 ± 0.48%AD/10^5^ cells at 2 h, and 3.76 ± 0.36%AD/10^5^ cells at 4 h (Figure 2B). The *K*_D_ of ^64^Cu-NOTA-Trodelvy-F­(ab’)_2_ was calculated to be 7.63 nM for NCI-N87 cells and 10.65 nM for BxPC3 cells (Figure 2C), indicating high specificity and strong affinity for Trop2. Furthermore, immunohistochemical staining was done to evaluate Trop2 expression in eight normal human tissue types, where lungs show obvious Trop2 expression (Figure 2D). The purpose of this IHC panel was to provide a representative reference for Trop2 distribution in selected normal tissues and to inform the biodistribution and safety considerations of Trop2-targeted imaging agents. It is important to note, however, that extensive literature has reported Trop2 expression in a broader spectrum of normal human tissues, including strong positivity in several epithelial organs, as demonstrated by Trerotola et al.^23^

### ImmunoPET Imaging and Biodistribution Studies in Gastric Cancer Models

Two subcutaneous gastric cancer xenograft models were established to represent high and low Trop2 expression using NCI-N87 and HGC-27 cell lines, respectively. Radiolabeled full-length Trodelvy was tested exclusively in the model with high Trop2 expression to compare the tumor uptake and pharmacokinetics with those of the radiolabeled Trodelvy-F­(ab’)_2_ fragments. As shown in the ImmunoPET images (Figure 3A), the uptake of ^64^Cu-NOTA-Trodelvy in NCI-N87 tumor increased over time and became evident at 12 h postinjection with an uptake value of 8.47 ± 2.26%ID/g. The highest tumor uptake value was 12.10 ± 3.10%ID/g, observed at the final time point (48 h postinjection) before sacrifice. On the other hand, the tumor uptake of ^64^Cu-NOTA-Trodelvy-F­(ab’)_2_ peaked at 12 h postinjection (Figure 3B), with values of 10.83 ± 2.76%ID/g and 3.33 ± 1.12%ID/g in gastric tumors with high and low Trop2 expression, respectively. Tumor uptake in HGC-27 was significantly lower than in NCI-N87 (*p* = 0.0110). In the IgG-F­(ab’)_2_ control group, NCI-N87 tumor uptake was significantly reduced (4.00 ± 1.16%ID/g, *p* = 0.0155). As expected, kidney uptake was significantly higher for the antibody fragments compared to the intact antibodies due to their smaller size, enabling rapid renal clearance and a much shorter circulation time.

**Figure 3:**
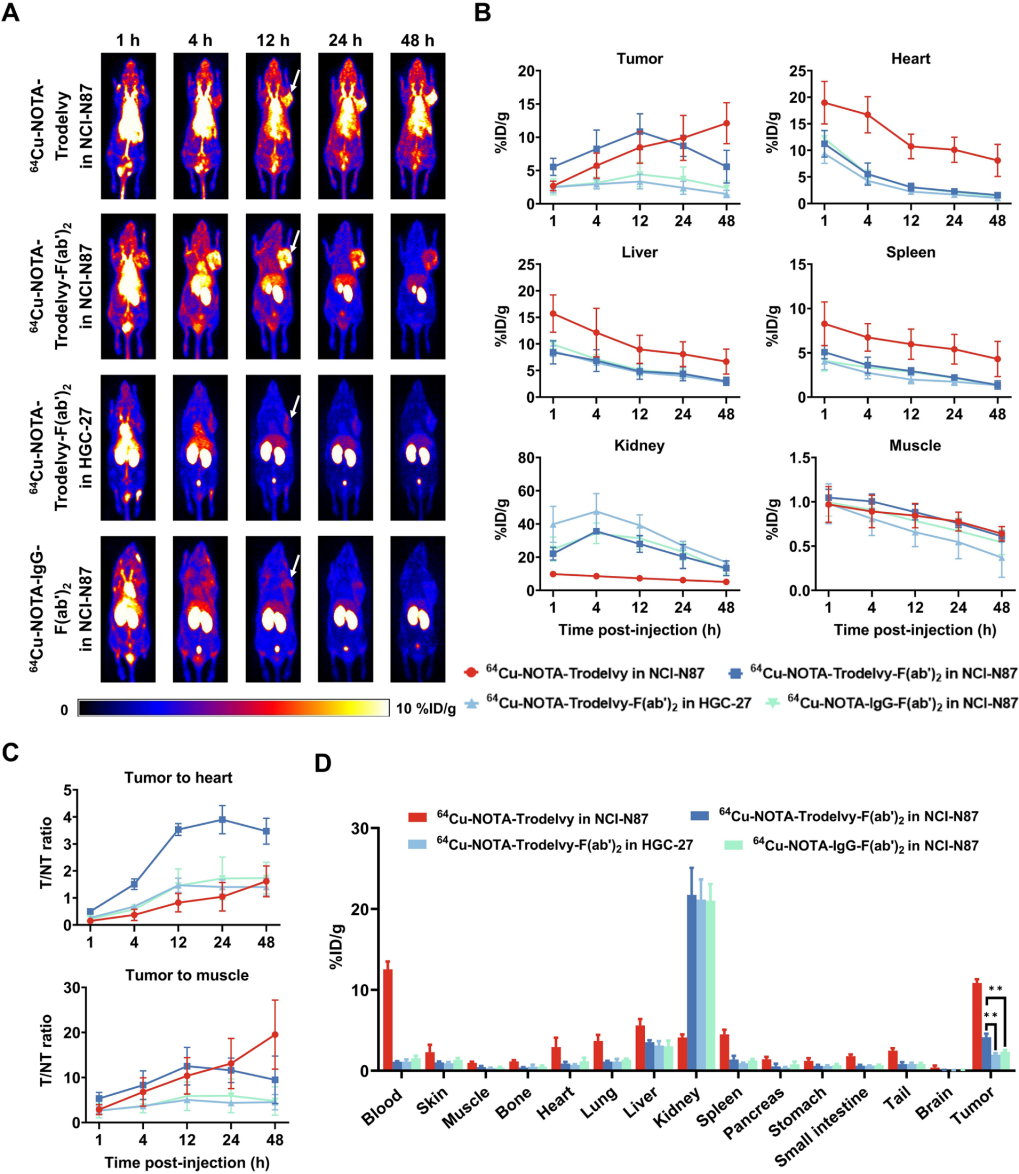
In vivo PET imaging and biodistribution of radiolabeled Trodelvy and F­(ab’)_2_ fragments in xenograft models of gastric cancer. (A) Representative PET images of mice bearing NCI-N87 and HGC-27 tumors at 1-, 4-, 12-, 24-, and 48 h postinjection of ^64^Cu-NOTA–Trodelvy, ^64^Cu-NOTA–Trodelvy-F­(ab’)_2_, or ^64^Cu-NOTA–IgG-F­(ab’)_2_. (B) Quantitative biodistribution data (%ID/g) of radiotracers in tumor and major organs over time. (C) Tumor-to-heart and tumor-to-muscle ratios over time as determined by region-of-interest image analyses. (D) Biodistribution of ^64^Cu-NOTA–Trodelvy, ^64^Cu-NOTA–Trodelvy-F­(ab’)_2_, or ^64^Cu-NOTA–IgG-F­(ab’)_2_ across major organs and tumors in mice bearing NCI-N87 and HGC-27 xenografts.

At 12 h postinjection, ^64^Cu-NOTA–Trodelvy-F­(ab’)_2_ demonstrated significantly higher target-to-nontarget (T/NT) ratios (Figure 3C) compared to ^64^Cu-NOTA–Trodelvy, indicating improved tumor targeting and specificity. The tumor-to-heart (T/H) ratio in NCI-N87 tumors reached 3.53 ± 0.22 for Trodelvy-F­(ab’)_2_, markedly higher than that of full-length Trodelvy (0.83 ± 0.34, *p* = 0.0003). In the HGC-27 group, the T/H ratio was 1.47 ± 0.27 (*p* = 0.0005), while the IgG-F­(ab’)_2_ control in NCI-N87 tumors yielded a significantly lower T/H ratio of 1.32 ± 0.39 (*p* = 0.0010). Similarly, the tumor-to-muscle (T/M) ratio for Trodelvy-F­(ab’)_2_ reached 12.51 ± 4.13 in NCI-N87 group, significantly higher than in HGC-27 group (5.03 ± 0.63, *p* = 0.0374). IgG-F­(ab’)_2_ group had a reduced T/M ratio of 4.92 ± 1.57 (*p* = 0.0420) in NCI-N87. However, the intact antibody yielded a comparable T/M ratio as Trodelvy-F­(ab’)_2_ in NCI-N87 group (10.54 ± 0.03, *p* = 0.7447).

*Ex vivo* biodistribution studies (Figure 3D) 48 h postinjection further confirmed the specificity of ^64^Cu-NOTA-Trodelvy-F­(ab’)_2_ for Trop2-positive tumors. In NCI-N87 tumors, ^64^Cu-NOTA-Trodelvy-F­(ab’)_2_ accumulation was 4.14 ± 0.44%ID/g, significantly higher than in the negative control group (2.00 ± 0.17%ID/g, *p* = 0.0014) and IgG-F­(ab’)_2_ group (2.35 ± 0.22%ID/g, *p* = 0.0031). Overall, these results demonstrate that both tracers showed Trop2-specific tumor targeting. While ^64^Cu-NOTA-Trodelvy exhibits superior tumor retention over time, ^64^Cu-NOTA-Trodelvy-F­(ab’)_2_ provides improved tumor-to-background contrast at earlier imaging time points.

### ImmunoPET Imaging and Biodistribution Studies in Pancreatic Cancer Models

Two subcutaneous pancreatic cancer models were established using BxPC3 and AsPC1 cell lines to represent high and low Trop2 expression, respectively. As shown in the ImmunoPET images (Figure 4A), uptake of ^64^Cu-NOTA-Trodelvy in BxPC3 tumors increased over time, becoming clearly visible at 12 h postinjection, with a measured uptake of 8.47 ± 1.25%ID/g. The highest tumor uptake, 13.87 ± 2.59%ID/g, was observed at the final imaging time point prior to sacrifice. On the other hand, the tumor uptake of ^64^Cu-NOTA-Trodelvy-F­(ab’)_2_ peaked at 12 h postinjection (Figure 4B), with values of 10.00 ± 0.89 in BxPC3 tumors and 3.13 ± 0.96%ID/g in AsPC1 tumors. The uptake in HGC-27 tumors was significantly lower than in NCI-N87 tumors (*p* = 0.0008). Similarly, IgG-F­(ab’)_2_ showed significantly lower uptake in NCI-N87 tumors (3.23 ± 0.32%ID/g, *p* = 0.0002).

**Figure 4:**
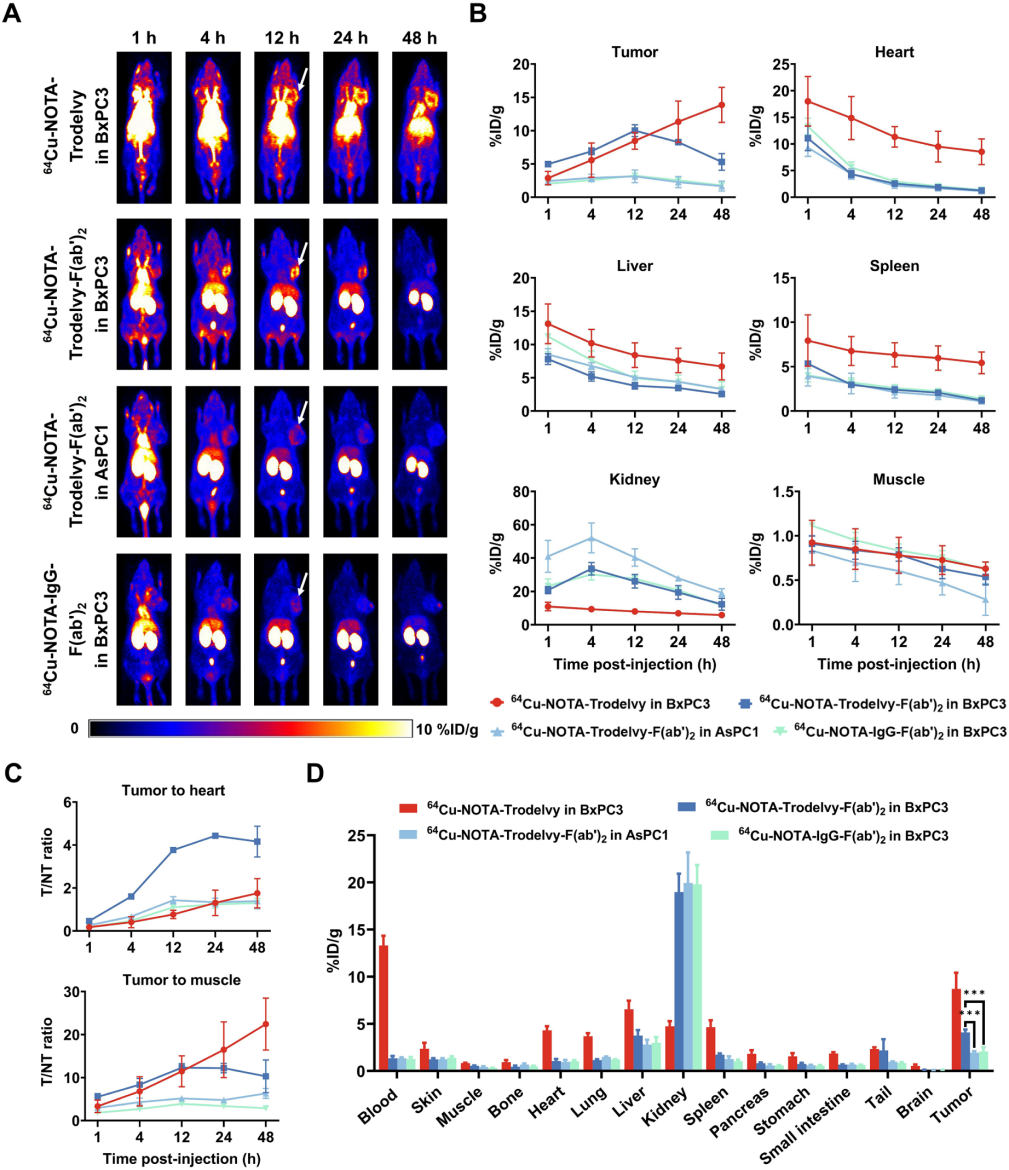
In vivo PET imaging and biodistribution of radiolabeled Trodelvy and F­(ab’)_2_ fragments in xenograft models of pancreatic cancer. (A) Representative PET images of mice bearing Bxpc3 and AsPC1 tumors at 1-, 4-, 12-, 24-, and 48 h postinjection of ^64^Cu-NOTA–Trodelvy, ^64^Cu-NOTA–Trodelvy-F­(ab’)_2_, or ^64^Cu-NOTA–IgG-F­(ab’)_2_. (B) Quantitative biodistribution data (%ID/g) of radiotracers in tumor and major organs over time. (C) Tumor-to-heart and tumor-to-muscle ratios over time as determined by region-of-interest image analyses. (D) Biodistribution of ^64^Cu-NOTA–Trodelvy, ^64^Cu-NOTA–Trodelvy-F­(ab’)_2_ or ^64^Cu-NOTA–IgG-F­(ab’)_2_ across major organs and tumors in mice bearing Bxpc3 and AsPC1 xenografts.

At 12 h postinjection, ^64^Cu-NOTA–Trodelvy-F­(ab’)_2_ demonstrated significantly higher T/NT ratios (Figure 4C) compared to ^64^Cu-NOTA–Trodelvy, indicating improved tumor targeting and specificity. The T/H ratio in BxPC3 tumors reached 3.77 ± 0.07 with Trodelvy-F­(ab’)_2_, markedly higher than that of full-length Trodelvy (0.77 ± 0.19, *P* < 0.0001). The AsPC1 group showed reduced uptake (1.43 ± 0.16, *P* < 0.0001), while the IgG-F­(ab’)_2_ group showed significantly lower T/H ratios in NCI-N87 tumors (1.10 ± 0.06, *P* < 0.0001). Similarly, the T/M ratio for Trodelvy-F­(ab’)_2_ in BxPC3 group was 12.29 ± 0.91, significantly higher than in AsPC1 group (5.16 ± 0.31, *P* = 0.0002). In the IgG-F­(ab’)_2_ group, the T/M ratio in NCI-N87 tumors was reduced to 3.88 ± 0.12 (*P* = 0.0420). However, the intact antibody yielded a comparable T/M ratio to Trodelvy-F­(ab’)_2_ in the NCI-N87 group (10.54 ± 0.03, *P* < 0.0001).

*Ex vivo* biodistribution studies (Figure 4D) confirmed significantly higher tumor uptake of ^64^Cu-NOTA-Trodelvy-F­(ab’)_2_ in BxPC3 xenografts compared to control and IgG-F­(ab’)_2_ groups. At 48 h p.i., uptake of ^64^Cu-NOTA-Trodelvy-F­(ab’)_2_ in BxPC3 tumors was 4.10 ± 0.31%ID/g, while uptake in the negative control and IgG-F­(ab’)_2_ groups was significantly lower (1.95 ± 0.15%ID/g, *P* = 0.0004; 2.08 ± 0.45%ID/g, *P* = 0.0004, respectively). Overall, these results demonstrate that both tracers showed specific Trop2 binding.

### Histological and Immunohistochemical Analysis of Tumor and Normal Tissues

Hematoxylin and eosin (H&E) staining of tumor xenografts derived from the gastric and pancreatic cancer cell lines confirmed viable tumor morphology, with no signs of necrosis of hemorrhage (Figure 5A). Immunohistochemical (IHC) staining further validated Trop2 expression patterns: NCI-N87 and BxPC3 tumors exhibited strong positive staining, consistent with their known Trop2-positive status (Figure 5B), while HGC-27 and AsPC1 tumors showed minimal to undetectable staining, confirming their Trop2-negative phenotype. To assess potential off-target expression and systemic toxicity, we further examined major organs by H&E and IHC staining. Histological examination showed unchanged tissue architecture across various major organs with no signs of inflammation or pathological damage following radiotracer administration (Figure 5C). IHC staining using a Trop2-specific antibody (clone EPR20043), validated for murine tissues, revealed detectable Trop2 expression in the lung. This observation aligns with previously reported Trop2 expression patterns in normal human tissues and may inform potential off-target tracer uptake (Figure 5D).

**Figure 5:**
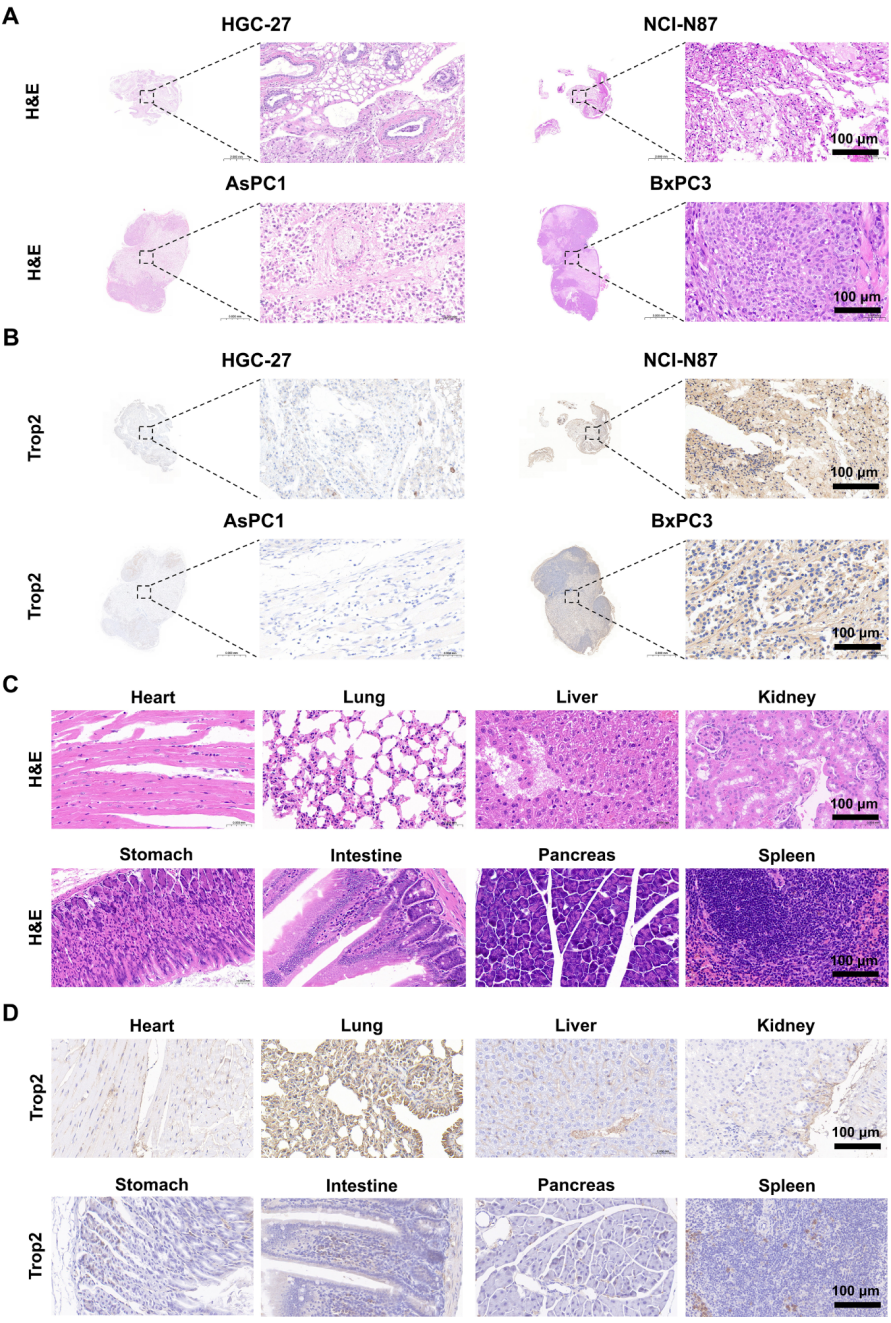
Histological and immunohistochemical evaluation of Trop2 expression in tumor and normal tissues. (A) H&E staining of tumor sections from four cancer cell line-derived xenografts. (B) Immunohistochemical staining (IHC) for Trop2 expression in tumor tissues. (C) H&E and (D) IHC staining of various major organs.

### Immunofluorescence Staining of Tumor and Normal Tissues

Immunofluorescence staining was performed to assess vascularization (CD31) and Trop2 expression in both tumor and normal tissues. Tumors derived from NCI-N87 and BxPC3 xenografts showed abundant CD31-positive vasculature along with strong Trop2 expression, consistent with their Trop2-positive phenotype. In contrast, HGC-27 and AsPC1 tumors exhibited weaker staining for both markers (Figure 6A), reflecting their lower vascular density and Trop2 expression levels. In normal organs (Figure 6B), CD31 staining revealed well-defined vascular structures across all major organs, while some Trop2 expression was detected in the lung, consistent with prior immunohistochemistry results. Trop2 expression in all other major organs was negligible compared to tumor tissues.

**Figure 6:**
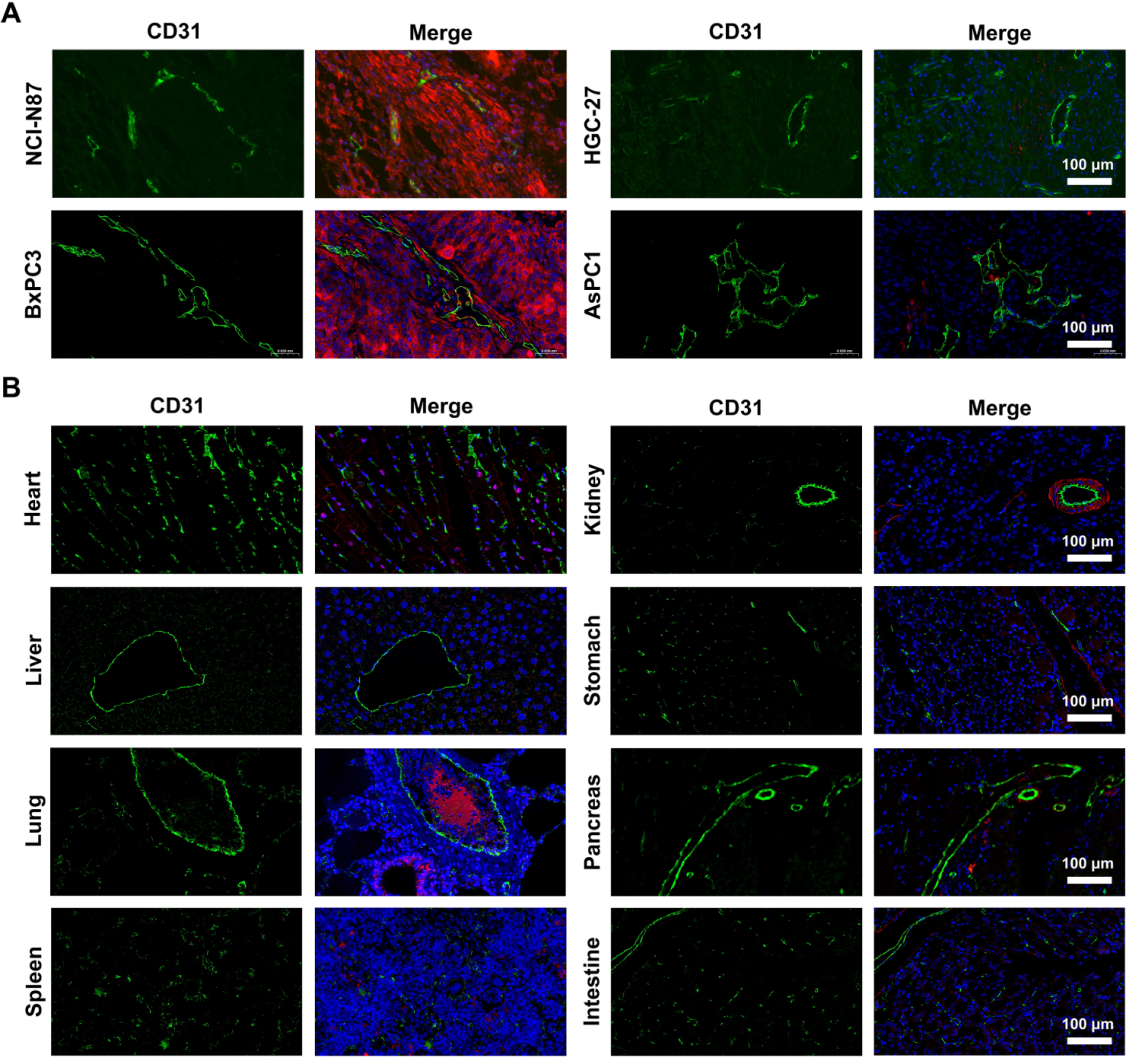
Immunofluorescence staining of Trop2 and vasculature in tumor and normal tissues. (A) Staining of CD31 (green), Trop2 (red), and nuclei (blue) in tumor sections from xenografts derived from gastric and pancreatic cancer cell lines and (B) in various major organs.

### Radiation Dosimetry Extrapolation to Humans

The radiation dose estimates for human organs, extrapolated from murine biodistribution data, are presented in Table S1, Supporting Information. The estimated systemic effective dose for an adult woman was 0.0273 mSv/MBq, which falls within the acceptable range for conventional nuclear medicine procedures. These findings support the translational potential of the radiotracer for clinical imaging applications.

## Discussion

ImmunoPET represents a cutting-edge molecular imaging modality, offering precise, noninvasive visualization of antigen-specific interactions in vivo. This technique has emerged as a promising modality for assessing Trop2 expression in real time, enabling patient stratification for targeted therapies and longitudinal monitoring of therapeutic response.^15^ However, the clinical translation of ImmunoPET has been constrained by the pharmacokinetic limitations of full-length antibodies.

Intact monoclonal antibodies (∼150 kDa) exceed the renal filtration threshold and are subject to FcRn-mediated recycling, resulting in prolonged circulation half-life.^24,25^ These properties delay optimal imaging windows and increase background signal, thereby diminishing image contrast and diagnostic efficiency. To address these limitations, antibody fragments such as F­(ab’)_2_ have gained attention due to their favorable pharmacokinetics.

F­(ab’)_2_ fragments, produced through enzymatic cleavage of the parent IgG antibody, retain antigen-binding capacity while lacking the Fc region. This structural modification enables rapid blood clearance, reduced off-target accumulation, and lower immunogenicity, making them ideal for molecular imaging applications that do not require immune effector functions.

Prior research has underscored the diagnostic value of Trop2-targeted imaging. For instance, Chen et al.^26^ reported the use of an ^89^Zr-labeled anti-Trop2 monoclonal antibody (DFO-AF650), which achieved a tumor uptake value of 28.8 ± 7.63%ID/g in BxPC3 pancreatic xenografts at 120 h. The tracer also demonstrated excellent correlation between PET images and biodistribution data in both subcutaneous and orthotopic models. Despite its robust performance, the long half-life of ^89^Zr and the prolonged retention of intact antibodies raise safety and feasibility concerns for routine clinical use. Building upon these findings, Li et al.^27^ developed a dual-labeled Trop2-specific antibody (IMB1636) for both imaging and radioimmunotherapy. The ^64^Cu-labeled version yielded substantial tumor uptake (8.95 ± 1.07%ID/g) at 48 h postinjection in T3M-4 xenografts, while the therapeutic isotope ^177^Lu achieved significant tumor suppression, validating its theranostic potential. Nevertheless, the slow pharmacokinetics of full antibodies necessitate extended imaging windows and increase exposure to radiation, limiting their clinical practicality.

Recently, Huang et al.^28^ developed and clinically validated novel nanobody-based PET imaging probes (^68^Ga-NOTA-T4 and ^68^Ga-NOTA-T5). In T3M-4 pancreatic tumor xenograft models, ^68^Ga-NOTA-T4 outperformed ^68^Ga-NOTA-T5 with significantly higher tumor uptake (4.60 ± 1.42%ID/g vs 2.10 ± 0.59%ID/g, *p* = 0.018) and improved tumor-to-organ contrast. In a clinical study involving ten patients, ^68^Ga-NOTA-T4 successfully identified Trop2-positive, Trop2-negative, and heterogeneous lesions, outperforming ^18^F-FDG in both specificity and background signal contrast. Unlike nanobodies, which are often developed de novo, F­(ab’)_2_ fragments can be directly derived from clinically approved antibodies such as Trodelvy, offering a streamlined path to clinical translation. Their bivalent binding and intermediate pharmacokinetics make them particularly well-suited for imaging applications that require both high target affinity and optimal imaging windows within 12–24 h. Future comparative studies evaluating F­(ab’)_2_, nanobodies, and other engineered antibody formats (e.g., scFv, diabodies) would be highly valuable for optimizing imaging performance and expanding clinical utility.

These limitations highlight the necessity of exploring alternative formats and isotopes that can optimize tumor visualization while reducing off-target effects. Antibody fragments such as F­(ab’)_2_, when combined with medium-lived radionuclides like ^64^Cu, offer a balanced solution—providing sufficient biological half-life for target accumulation while enabling same-day imaging and minimizing radiation burden.^29,30^ This approach may significantly enhance the clinical viability of Trop2-targeted ImmunoPET and expand its utility in precision oncology.

The ∼100 kDa F­(ab’)_2_ format shortens blood residence and enhances early tumor-to-background contrast (notably T/H at ∼12 h p.i.), but—as expected—incurs higher renal activity from glomerular filtration and residualizing ^64^Cu catabolites. In our models, this pharmacokinetic balance enabled practical same-day imaging with high contrast, while histology showed no renal pathology and human dosimetry estimates remained within conventional research limits. Prospective mitigation strategies (e.g., amino-acid/gelofusine coinfusion, chelator/format tuning, or nonresidualizing labels) merit evaluation in future translational studies.

Beyond intact mAbs, multiple Trop2-targeting nanobody tracers have shown promising preclinical and early clinical performance. In this work we selected an F­(ab’)_2_ fragment to balance manufacturability, binding, kinetics, and translational risk. F­(ab’)_2_ can be generated rapidly and reproducibly from a humanized, approved antibody (Trodelvy) using IdeS cleavage and bead-based separations, obviating de novo discovery and extensive engineering. The bivalent F­(ab’)_2_ preserves avidity while accelerating blood clearance relative to intact IgG, enabling practical “same-day” imaging with high tumor-to-background ratios (∼12 h p.i.). In our models the tracer leading to strong and selective tumor uptake and favorable image contrast compared with the intact antibody at early time points. We recognize that nanobodies provide ultrarapid imaging and excellent tissue penetration; however, their monovalency, very high renal uptake, and the need for humanization may complicate abdominal readouts and translation. Overall, F­(ab’)_2_-based Trop2 immunoPET represents a pragmatic, patient-stratification tool that complements nanobody approaches; prospective comparisons will help optimize format selection for specific clinical contexts.

In this study, we confirmed that both full-length Trodelvy and its F­(ab’)_2_ derivative display comparable antigen-binding characteristics, as demonstrated by flow cytometric analysis and confocal imaging. The antibody fragment, engineered through enzymatic cleavage, exhibited enhanced pharmacokinetic properties due to its reduced molecular weight and lack of Fc domain. These structural advantages led to rapid renal elimination *in vivo*, thereby enabling precise and timely visualization of Trop2 expression in tumors. Notably, the early tumor accumulation of ^64^Cu-labeled Trodelvy-F­(ab’)_2_—facilitated by efficient tissue penetration—supports its application for same-day imaging, which holds substantial promise for clinical workflows requiring rapid diagnostics.

The accelerated blood clearance profile of the F­(ab’)_2_ tracer significantly improved image contrast between tumor and nontumor tissues. In animal models, blood retention of the F­(ab’)_2_ conjugate decreased more sharply over time compared to the intact antibody, which demonstrated slower elimination. Specifically, in NCI-N87 and BxPC3 xenograft-bearing mice, the tracer concentration in circulation dropped to minimal levels within 48 h, further supporting its value in enhancing signal-to-background ratios. These findings establish the fragment-based imaging agent as a sensitive and selective tool for delineating Trop2-positive tumors with improved clarity.

## Conclusion

In conclusion, this investigation led to the successful design and validation of a radiochemically stable, highly purified ^64^Cu-labeled NOTA-conjugated F­(ab’)_2_ fragment of Trodelvy, tailored for immunoPET applications targeting Trop2-expressing tumors. Through comprehensive evaluation in murine models of gastric and pancreatic malignancies, the F­(ab’)_2_-based imaging agent exhibited distinct advantages over its full-length antibody counterpart. These included retained Trop2-binding specificity and affinity, accelerated accumulation within tumor tissue, and markedly enhanced contrast relative to nontarget regions. The smaller size and faster clearance kinetics of the F­(ab’)_2_ construct contributed to its favorable biodistribution profile, enabling more efficient imaging within clinically relevant timeframes.

## Supplementary Material



## Data Availability

The data sets generated during and analyzed during the current study are available from the corresponding author on reasonable request.

## Notes

Methodological details and additional data that reinforce the results of this research; in-depth explanation of cell culture conditions, the creation of animal models, methods for histological analysis, and statistical techniques (PDF)
