# Supplementary material for: Noninvasive Evaluation of Trop2 Expression Using 64Cu-NOTA-Trodelvy-F(ab’)2 in Gastric and Pancreatic Cancer
Source: Mol Pharm. 2025 Nov 7;22(12):7630–40. doi: 10.1021/acs.molpharmaceut.5c01223 (PMC12673595; doi:10.1021/acs.molpharmaceut.5c01223)
Supplement: Supplementary file 1 [file mp5c01223_si_001.pdf]

# Noninvasive evaluation of Trop2 expression using $^{64}$Cu-NOTA-Trodelvy-F(ab')$_2$ in gastric and pancreatic cancer

Wenpeng Huang [1,2#], Fangfang Chao [3#], Ruobing Li [4], Yihan Yang [1], Jessica C. Hsu [2], Molly C. DeLuca [2], Jonathan W. Engle [2], Xingmin Han [3*], Lei Kang [1*], Weibo Cai [2*]

[#]Wenpeng Huang and Fangfang Chao contributed equally to this work.

1. Department of Nuclear Medicine, Peking University First Hospital, Beijing, 100034, China
2. Departments of Radiology and Medical Physics, University of Wisconsin - Madison, Madison, Wisconsin 53705, USA
3. Department of Nuclear Medicine, The First Affiliated Hospital of Zhengzhou University, Zhengzhou 450052, Henan Province, China
4. College of Letters and Science, University of Wisconsin – Madison, Madison, Wisconsin 53705, USA

***Corresponding author:**

**Weibo Cai**, E-mail: wcai@uwhealth.org

**Lei Kang**, E-mail: kanglei@bjmu.edu.cn

**Xingmin Han**, Email: xingminhan@126.com

**Supplementary materials and methods**

**Preparation of F(ab')$_2$ fragments**

Trodelvy was kindly provided by the Department of Urology at Peking University First Hospital. Trodelvy-F(ab')$_2$ fragments were prepared using the Immunoglobulin-degrading Enzyme from Streptococcus pyogenes (IdeS) protease kit (V7511, Promega). A total of 5 mg of Trodelvy (10 mg/mL) was incubated with IdeS protease in digestion buffer (50 mM sodium phosphate, 150 mM NaCl, pH 6.6) for 60 minutes at 37°C with moderate shaking (650 rpm) on a ThermoShaker (Biosan, USA).

To prepare Magne® Protein A Beads (G7471, Promega), 120 μL of the bead suspension was gently vortexed or inverted to ensure uniformity. The storage buffer was removed using a magnetic stand, and the beads were washed twice with 500 μL of PBS. The digested sample was then incubated with the Magne® Protein A Beads for 1 h on an end-over-end mixer (ThermoMixer system), ensuring the beads remained in suspension throughout. The Fc fragment bound to the beads was separated as sediment, while the purified F(ab')$_2$ fragments were collected in the supernatant using a magnetic stand.

Subsequently, 100 μL of MagneHis™ Ni Particles (V8560, Promega) was combined with the Trodelvy/IdeS digested sample and incubated for 3 minutes at room temperature. The supernatant, containing the desired purified F(ab')$_2$ fragments, was carefully collected using a pipette. The per-batch recovery (mg) of 5 mg Trodelvy input was 3.1-3.5 mg.

The products were analyzed using non-reducing Sodium Dodecyl Sulfate-Polyacrylamide Gel Electrophoresis (non-reducing SDS-PAGE) and high-performance liquid chromatography (HPLC). Non-reducing SDS-PAGE was performed using 3–12% NativePAGE™ Bis-Tris Mini Protein Gels (BN1001BOX, Thermo Fisher, USA) at 100 V for 80 minutes. The gels were subsequently stained with Coomassie Brilliant Blue at room temperature, and the protein bands were visualized.

HPLC analysis was conducted on a Dionex UltiMate 3000 Chromeleon

system (Thermo Fisher Scientific) using a Yarra$^{TM}$ 3 μm SEC-4000 column (00H-4514-K0). The injection volume was 250 μg, and the retention time was 35 minutes. F(ab')$_2$ fragments of an isotype IgG (Thermo Fisher Scientific) were prepared as a control following the same procedure described above.

### Conjugation and radiolabeling

Conjugation of S-2-(4isothio-cyanatobenzyl)-1,4,7-triazacyclononane-1,4,7-triacetic acid (*p*-SCN-Bn-NOTA) (Macrocyclics, Plano, USA) was performed at pH 9.0 for 2–3 h, with a NOTA-to-Trodelvy and NOTA-to-Trodelvy-F(ab')$_2$ molar ratio of 15–20: 1. The resulting NOTA-Trodelvy and NOTA-Trodelvy-F(ab')$_2$ conjugates were purified using PD-10 columns, with PBS as the mobile phase, and the concentration was determined using a NanoDrop spectrophotometer (Thermo Scientific). $^{64}$Cu was produced by a PET trace cyclotron (GE Healthcare, Madison, WI) via the $^{64}$Ni(p,n)$^{64}$Cu nuclear reaction.

For radiolabeling, approximately 74 MBq (~2 mCi) of $^{64}$Cu was diluted added to 0.1 M sodium acetate buffer (pH 5.0) and incubated with NOTA-Trodelvy and NOTA-Trodelvy-F(ab')$_2$ (~200 μg) at 37°C for 60 minutes. The $^{64}$Cu-NOTA-Trodelvy and $^{64}$Cu-NOTA-Trodelvy-F(ab')$_2$ product was purified using a PD-10 column with PBS, and radioactive fractions containing the $^{64}$Cu-labeled conjugate were collected and filtered through a 0.2 μm syringe filter for *in vivo* applications. A similar protocol was used to prepare $^{64}$Cu-NOTA-IgG and $^{64}$Cu-NOTA-IgG-F(ab')$_2$. Labeling efficiency and radiochemical purity were assessed by radio thin-layer chromatography (Radio-TLC).

### Cell culture and animal model

The human gastric cancer cell lines NCI-N87 and HGC-27, along with the pancreatic cancer cell lines BxPC3 and AsPC1, were obtained from the American Type Culture Collection (ATCC, Manassas, VA, USA). NCI-N87, HGC-27, BxPC3, and AsPC1 cells were cultured in RPMI 1640 medium (Invitrogen, Carlsbad, CA) supplemented with 10% fetal bovine serum (FBS)

and maintained at 37°C in a 5% $CO_2$ incubator. Cells were utilized for *in vitro* or *in vivo* experiments once they reached 70%–90% confluence.

All applicable international, national, and/or institutional guidelines for the care and use of animals were followed. The study was approved by the Medical Ethics Committee of the First Affiliated Hospital of Zhengzhou University (2021-KY-1070-002), the Institutional Animal Care and Use Committee at the University of Wisconsin-Madison (#M005630), and the Animal Committee of Peking University First Hospital (No. J2023059). For tumor model establishment, four to five-week-old female Athymic Nude-Foxn1nu mice were procured from Envigo (Indianapolis, IN). Tumors were induced by subcutaneous injection of 4–5 × $10^6$ cells, suspended in a 70 μL mixture of PBS and Matrigel (1:1, Corning, USA), into the mice's front flank. Tumor growth was monitored every other day, with mice selected for *in vivo* experiments once tumor diameters reached 8–15 mm (approximately 2–3 weeks post-inoculation for NCI-N87 and HGC-27, and 3 weeks for BxPC3 and AsPC1).

**Flow cytometry**

All cells were washed twice with cold PBS and adjusted to a final concentration of 1.0 × $10^5$ cells/mL before analysis using the Lightning cytometer (ThermoFisher Attune). Incubation was performed using Trodelvy, Trodelvy-F(ab')$_2$, NOTA-Trodelvy, and NOTA-Trodelvy-F(ab')$_2$ at a final concentration of 50 μg/mL for 1 h on ice in the dark. Subsequently, the cells were incubated with Alexa Fluor 488 (AF488)-labeled rabbit anti-human secondary antibodies at room temperature for 0.5 h. Data acquisition and analysis of mean fluorescence intensities were performed using FlowJo software.

**Cell uptake experiments**

Cells were seeded in 24-well plates at a density of 2.0 × $10^5$ cells per well, followed by the addition of 1 mL of serum-free medium containing 37 kBq of

$^{64}$Cu-NOTA-Trodelvy and $^{64}$Cu-NOTA-Trodelvy-F(ab')$_2$. The plates were incubated at 37°C for varying time points (1h, 2 h, 4 h). After incubation, 1 mL of 1 M NaOH was added to each well for cell lysis, and the lysates were collected. The radioactivity in both the supernatants and lysates was measured using an automatic gamma counter (Wizard2, PerkinElmer).

**Cell binding assay**

For binding studies, NCI-N87 and BxPC3 cells were plated in a 96-well filter plate at a density of 1.0 × $10^5$ cells per well. A range of $^{64}$Cu-NOTA-Trodelvy and $^{64}$Cu-NOTA-Trodelvy-F(ab')$_2$ solutions with final concentrations between 0.15 and 150 nM were prepared, and cells were incubated with these solutions at 37°C for 4 h. After incubation, the cells were thoroughly washed, and radioactivity was measured using a gamma counter. Surface Trop2 expression, apparent dissociation constant ($K_D$), and maximum binding capacity ($B_{max}$) were determined using total and nonspecific binding data analyzed with GraphPad Prism software, with the $K_D$ value largely depends on the surface density of Trop2 expression on the cell system which is used for affinity measurements.

**ImmunoPET imaging**

ImmunoPET imaging studies were conducted using an Inveon microPET scanner designed for rodents (Siemens Medical Solutions USA, Inc.). Tumor-bearing mice received an intravenous injection of 7.4–11.1 MBq of $^{64}$Cu-NOTA-Trodelvy-F(ab')$_2$, and imaging was performed at 1, 4, 12, 24, and 48 h post-injection (p.i.). Images were calibrated to display signals within a range of 0–10% of the injected dose per volume, expressed as %ID/g. Quantitative analysis involved delineating regions of interest (ROI) in the heart, liver, kidneys, muscles, spleen, and tumors. Tumor-to-heart (T/H) and tumor-to-muscle (T/M) ratios were subsequently calculated to assess tracer distribution.

***Ex vivo* biodistribution**

After the final imaging session, mice were euthanized via carbon dioxide asphyxiation. Key organs and tissues, including the tumor, heart, liver, spleen, lungs, kidneys, stomach, intestines, tail, muscles, bones, brain, skin, and blood, were excised and weighed. The radioactivity of each sample was quantified using an automated gamma counter, and biodistribution was expressed as %ID/g.

**Radiation dosimetry prediction**

Balb/c mice (female, 4–5 weeks old) were divided into 4 groups (n = 3). Balb/c mice were then injected with 0.74 MBq of $^{64}$Cu-NOTA-Trodelvy and $^{64}$Cu-NOTA-Trodelvy-F(ab')$_2$ (100 μL) through the tail vein and sacrificed 1, 4, 24 and 48 h after injection. The extrapolated doses to adult females were calculated using decay corrected $^{64}$Cu biodistribution data. The dosimetry analysis was performed using the OLINDA/EXM dose-spherical model provided by the OLINDA/EXM software.

**Histological analysis**

Histological assessments included hematoxylin-eosin (H&E) staining, immunohistochemistry (IHC), and immunofluorescence. Immunofluorescent staining was conducted on tumor tissues and major organs, including the lungs, liver, spleen, stomach, pancreas, and kidneys, to evaluate Trop2 expression according to established protocols. Primary antibodies used were anti-human Trop2 (1:300, EPR20043, Abcam) and mouse anti-human CD31 (1:300, GB11063-1, Servicebio). Secondary antibodies consisted of AF488-labeled goat anti-rabbit IgG (1:200, A56021, Thermo Fisher Scientific) and Cy3-labeled goat anti-mouse IgG (1:200, Servicebio). Tissue morphology and staining patterns were analyzed and captured using a NIKON Eclipse Ti confocal microscope.

**Statistical analysis**

Quantitative data are expressed as mean ± standard deviation (SD). Statistical analyses were conducted using the *Student's t-test* or *one- or two-way ANOVA* in GraphPad Prism version 8.0. A P-value < 0.05 was considered statistically significant. All experiments were performed in triplicate or more, with results presented as mean ± SD unless specified otherwise. Statistical significance in the figures is denoted by asterisks: * $P < 0.05$; ** $P < 0.01$; *** $P < 0.001$; **** $P < 0.0001$.

## Supplementary results

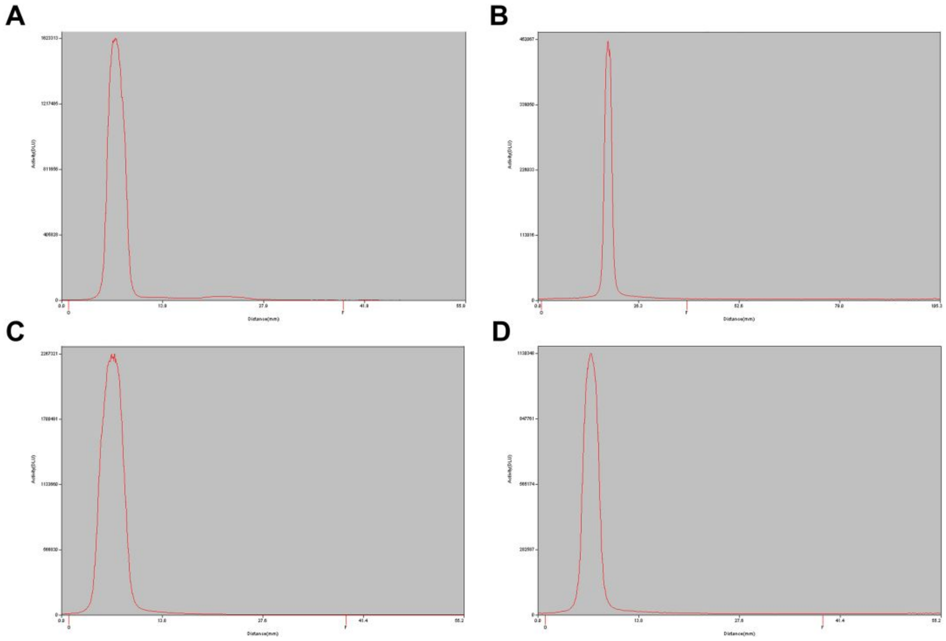


**Supplementary Fig. 1 Radio-TLC analysis of the radioactive assay utilizing $^{64}$Cu-NOTA-Trodelvy-F(ab')$_2$ as the substrate. (A)** Radiochemical yield of $^{64}$Cu-NOTA-Trodelvy-F(ab')$_2$. **(B)** Radiochemical purity of $^{64}$Cu-NOTA-Trodelvy-F(ab')$_2$ following purification with a PD-10 column. **(C–D)** Radiochemical purity of $^{64}$Cu-NOTA-Trodelvy-F(ab')$_2$ after 24 h of incubation in 0.01 M PBS and 5% human serum albumin.

## Radiation dosimetry extrapolation to humans

Dosimetry analysis was conducted using OLINDA/EXM software. Estimated human dosimetry was derived from average %ID/g values obtained from serial

PET scans on BALB/c mice, converted to %ID in humans. Assuming similar biodistribution in adult humans as in animal models, a monoexponential model was applied to the time–activity curves. OLINDA provides effective dose outputs, and weighting factors from the International Commission on Radiological Protection Publication 103 were utilized to convert these to absorbed doses for each organ. The radiation dose estimates for human organs, based on biodistribution data, are presented in **Table S1**. The estimated systemic effective dose for an adult woman was 0.0273 mSv/MBq, which falls within acceptable limits for conventional nuclear medicine research.

**Table S1. Human organ radiation dosimetry estimation of $^{64}$Cu-NOTA-Trodelvy-F(ab')$_2$.**

| Target Organ | mSv/MBq |
|---|---|
| Adrenals | 3.32E-04 |
| Brain | 5.44E-05 |
| Breasts | 3.62E-03 |
| Esophagus | 1.22E-03 |
| Eyes | 0.00E00 |
| Gallbladder Wall | 3.29E-04 |
| Left colon | 1.78E-03 |
| Small Intestine | 3.30E-04 |
| Stomach Wall | 4.12E-03 |
| Right colon | 1.79E-03 |
| Rectum | 8.66E-04 |
| Heart Wall | 1.86E-04 |
| Kidneys | 8.90E-04 |
| Liver | 5.44E-04 |
| Lungs | 1.91E-03 |
| Ovaries | 1.51E-03 |

| | |
|---|---|
| Pancreas | 1.51E-03 |
| Salivary Glands | 3.19E-04 |
| Red Marrow | 3.81E-03 |
| Osteogenic Cells | 2.90E-04 |
| Spleen | 1.41E-04 |
| Thymus | 3.07E-04 |
| Thyroid | 1.29E-03 |
| Urinary Bladder Wall | 1.34E-03 |
| Uterus | 1.74E-04 |
| Effective Dose | 2.73E-02 |
